# Supplementary material for: Hydrodynamic dispensing and electrical manipulation of attolitre droplets
Source: Nat Commun. 2016 Aug 12;7:12424. doi: 10.1038/ncomms12424 (PMC4990644; doi:10.1038/ncomms12424)
Supplement: Supplementary Information — Supplementary Figures 1-2, Supplementary Notes 1-2 and Supplementary Reference. [file ncomms12424-s1.pdf]

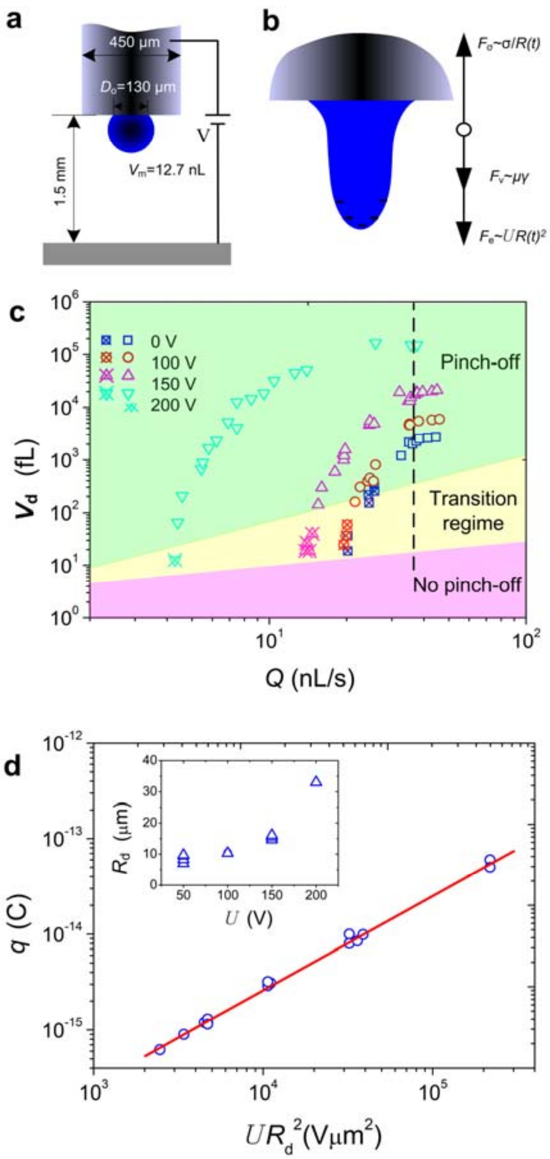

Supplementary Figure 1. Dispensing in electric field. a, geometry parameter setting during the experiments; **b**, illustration of the forces applied on the liquid column during drainage; **c**, Influence of applied potential and drainage rate on the volume of the droplets; **d**, influence of applied potential on the size and charge amount of the dispensed droplets.

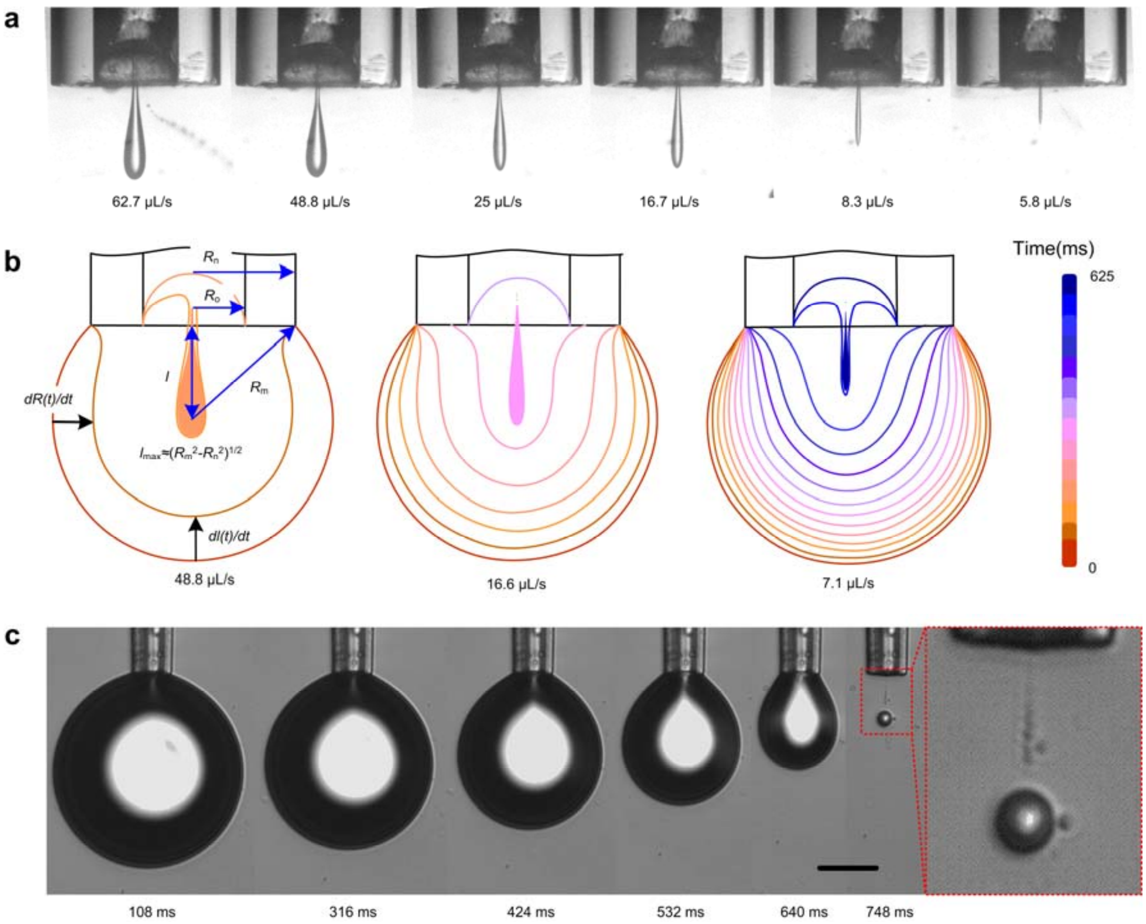

Supplementary Figure 2. a. Liquid columns at the moment of pinch off with different drainage rate; **b.** Evolution process of the sessile drops with different drainage rate. The profiles of the mother drop were obtained from the high speed images. The time between adjacent profiles is 48 ms; **c.** Deformation process of the sessile drop when its radius is much larger than the orifice. Scale bar, 50 μm.

Supplementary Note 1. Dispensing in an electric field

After the application of an electric field, the deformation of the sessile drop during the drainage is also affected by electrophoretic force F_e besides viscous force F_v. An analytical solution of F_e is challenging since the charge distribution during the drainage changes dynamically. Nonetheless, we note that F_e scales as $F_e \sim qE$, where q is the charge distributed on the liquid column (mainly distributed on the column peak), E is the electric field strength and scale as $E \sim U$, where U is the potential. q scales as $q \sim ER(t)^2 \sim UR(t)^2$. $R(t)$ is the radius of the liquid column during drainage. Therefore, F_e should scales as $F_e \sim U^2R(t)^2$. There is a positive feedback between $R(t)$ and F_e.

Supplementary Figure 1a shows the geometric parameters when dispensing droplets in electric field. Supplementary Figure 1b shows the force analysis of the liquid column during the drainage process. Supplementary Figure 1c shows the influence of U and Q on the volume of the daughter droplet V_d. The drop volume V_d increases with increasing U for otherwise constant parameters. According to Hamlin et al. [1], the charge distributed at the apex of the drop stretches the drop during the shrinkage process and accelerates the break-up of the neck. Therefore, more liquid is left in the daughter droplet. The experimental results indicate that the electric field can significant enlarge the size of the daughter droplets.

In order to provide a quantitatively analysis, the charge for 17 droplets is measured when drainage rate is between 32 nL/s and 38 nL/s (points near the vertical dashed line in Supplementary Figure 1c) at various applied potentials. The charges of the droplets are estimated by their moving velocity after pinch-off. This method was also used Hamlin et al. [1]. Supplementary Figure 1d shows the relationship between q and UR_d^2. We see excellent agreement with the prediction of the scaling laws. The inset of Supplementary Figure 1d shows the influence of U on R_d for the same17 droplets.

Supplementary Note 2 Explanation of the plateau phenomenon shown in Fig. 4c

As illustrated in Fig. 4c, R_d cannot grow unlimited with $(\mu_2 Q/\sigma_{12})^{1/2}$. Here the analysis of the experimental data in Fig. 4c is detailed. Supplementary Figure 2a shows the shape of the liquid column at the moment of pinch-off. When Q is higher than 48.8 μL/s, the radius and height of the column do not show much difference. For instance, the sessile drop contracts much faster when Q is 62.7 μL/s, the liquid column just before pinch-off does not appear very different from the drainage rate of 48.8 μL/s (Supplementary Figure 2a). Supplementary Figure 2b shows the deformation process of the sessile drop.

A full description of the shape of the liquid column is challenging due to the many factors influencing the drainage process. However, with increasing drainage rate Q, the difference between horizontal shrinking rate $dR(t)/dt$ and the vertical shrinking rate $dl(t)/dt$ becomes smaller. In the case of higher Q, the horizontal shrinking rate $dR(t)/dt$ is close to the vertical shrinking rate $dl(t)/dt$. This suggests that the maximum length of liquid column l_{max} should be smaller than $(R_m- R_n)^{1/2}$ which is about 660 μm under the particular experimental conditions (Supplementary Figure 2b). If the sessile drop is in contact with the inner wall of the nozzle, l_{max} should be smaller than $(R_m- R_o)^{1/2}$. According to the classical theory of Rayleigh-plateau instability, the critical condition for the instability of a liquid column is its extension factor ε, defined as the ratio of its length l to circumference πR_c. It should be larger than 1. Therefore, $R_{c,\, max}$ should be smaller than l_{max}/π which is about 210 μm under the particular experimental conditions. The maximum radius of the droplet $R_{d,\, max}$ can be estimated after knowing l_{max} and $R_{c,\, max}$ which should be about 280 μm if residual drainage is neglected. The above analysis shows that there should be a maximum value $R_{d,\, max}$ for each particular experimental conditions. However, after the formation of the column, the residual drainage will further thin the column and finally result the pinch off. On the other hand, the above estimation is conducted under the assumption $\varepsilon = 1$. However, the liquid column has an extension factor ε between 4 and 10 (depending on the drainage rate), i.e. much larger than 1 (Supplementary Figure 2a). The combined action of the residual drainage and the high extension factor lead to the observed plateau value (about 150 μm) which is much smaller than $R_{d,\, max}$. In Fig. 4f, the

plateau is not observed because R_d is much smaller than its $R_{d, max}$ = 53 μm according to the geometry of the system.

The above analyses illustrate R_d is constrained by R_o and R_m. It also suggests that it should be possible to obtain daughter droplets similar or even larger than the orifice diameter if R_m is sufficiently large. Our experiments verified this prediction (Supplementary Figure 2c).

Supplementary Reference

1. Hamlin, B.S., Creasey, J.C. & Ristenpart, W.D. Electrically tunable partial coalescence of oppositely charged drops. *Phys. Rev. Lett.* **109**, 094501 (2012).
